# Health disparities among older adults following tropical cyclone exposure in Florida

**DOI:** 10.1038/s41467-023-37675-7

**Published:** 2023-04-19

**Authors:** K. Burrows, G. B. Anderson, M. Yan, A. Wilson, M. B. Sabath, J. Y. Son, H. Kim, F. Dominici, M. L. Bell

**Affiliations:** 1 Institute at Brown for Environment and Society, Brown University, Providence, RI USA; 2 Department of Environmental and Radiological Health Sciences, Colorado State University, Fort Collins, CO USA; 3 School of Ecology and Environment, Beijing Technology and Business University, Beijing, China; 4 Department of Statistics, Colorado State University, Fort Collins, CO USA; 5 T.H. Chan School of Public Health, Harvard University, Boston, MA USA; 6 School of the Environment, Yale University, New Haven, CT USA; 7 Division of Environmental and Occupational Health Sciences, School of Public Health, IL Chicago, USA

**Keywords:** Risk factors, Environmental impact, Public health

## Abstract

Tropical cyclones (TCs) pose a significant threat to human health, and research is needed to identify high-risk subpopulations. We investigated whether hospitalization risks from TCs in Florida (FL), United States, varied across individuals and communities. We modeled the associations between all storms in FL from 1999 to 2016 and over 3.5 million Medicare hospitalizations for respiratory (RD) and cardiovascular disease (CVD). We estimated the relative risk (RR), comparing hospitalizations during TC-periods (2 days before to 7 days after) to matched non-TC-periods. We then separately modeled the associations in relation to individual and community characteristics. TCs were associated with elevated risk of RD hospitalizations (RR: 4.37, 95% CI: 3.08, 6.19), but not CVD (RR: 1.04, 95% CI: 0.87, 1.24). There was limited evidence of modification by individual characteristics (age, sex, or Medicaid eligibility); however, risks were elevated in communities with higher poverty or lower homeownership (for CVD hospitalizations) and in denser or more urban communities (for RD hospitalizations). More research is needed to understand the potential mechanisms and causal pathways that might account for the observed differences in the association between tropical cyclones and hospitalizations across communities.

## Abstract (web summary)

Tropical cyclones pose a significant threat to human health. Here, authors show associations between tropical cyclones and risk of cause-specific hospitalizations, with elevated risks according to neighborhood-level poverty, homeownership and urbanicity.

## Introduction

Tropical cyclones (referred to as hurricanes in the North Atlantic, central North Pacific, and Eastern North Pacific Oceans) occur regularly across much of the globe. In the United States, tropical cyclones (TCs) were responsible for a higher number of deaths between 1980 and 2020 than any other weather-related disaster^1^. It is well established that TCs can impact morbidity and mortality through direct physical trauma (e.g., drowning or injury)^2^. There is mounting evidence that cyclones also have indirect impacts on health, often via infrastructure damage and healthcare disruptions^3^. In this study, we focus on the effects of TCs on cardiovascular and respiratory diseases. Previous studies have posited that the risk of cardiovascular events following storms may be attributed to increased stress^4^, changes in physical activity^5^, or delays in access to medical care^6^. Similar mechanisms have been proposed for respiratory diseases (including stress^7^ and delayed access to care^8^), but others may include power outages, which can impact ventilators and other electrically operated medical equipment^9^, or the formation of mold spores^10^. The research quantifying these risks has primarily focused on case studies of single storms, though recent work has begun to assess the simultaneous effects of multiple storms to better capture the effects of TCs on hospitalizations^11^ and preterm birth^12^.

While the evidence linking TCs and morbidity is growing, there remains a need to better understand which subgroups are most at-risk. Previous research has identified that the health effects of TCs are not uniform across populations, varying by age and race^13^ and community-level income^14^. However, this work remains limited and mostly concentrated on the impact of single major storms (e.g., Hurricane Katrina in New Orleans, 2005) and therefore may not be representative of all TCs^13^. Better understanding and identification of at-risk groups are essential for improved disaster preparedness.

In this paper, we address this gap in the literature by quantifying whether and how the association between TCs and daily Medicare hospitalizations for cardiovascular and respiratory diseases varied in Florida (FL), from 1999 to 2016 based on individual- (age group, sex, and Medicaid eligibility, as an indicator of socioeconomic status) and community-level (education, income, primary language, housing, urbanicity, and racial composition) characteristics. We focus particularly on variation in health risks across older adults (≥65 years of age), who comprise a high-risk group during environmental disasters due in part to restricted mobility and pre-existing health issues^15^.

## Results

### Tropical cyclone exposure

**Fig. 1:**
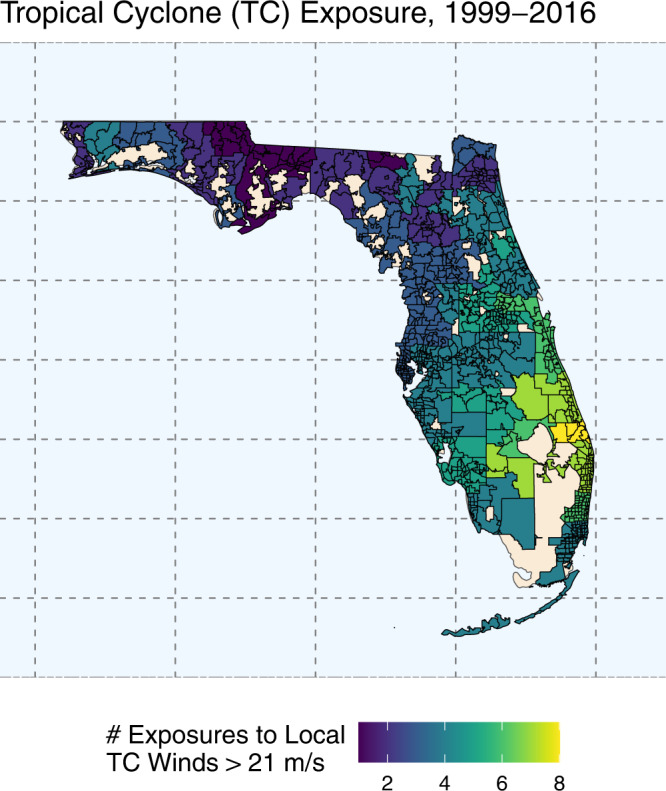
Tropical cyclone exposure in Florida at the zip code tabulation area (ZCTA) level (*n* = 983) from 1999 to 2016. Tan colored areas represent uninhabited areas (parks, bodies of water, etc.), or ZCTAs with no tropical cyclone exposure during the study period. Source data provided as a Source Data file.

In total, there were 20 unique storms that made landfall (or close approach) across the zip code tabulation areas (ZCTAs) in Florida from 1999 to 2016. ZCTA-level TC exposures are shown in Fig. 1. These storms resulted in a total of 3996 TC-exposed days across the 983 ZCTAs. Each ZCTA had an average of 4.07 (±1.54) storm\s over the 18-year study period, ranging from 1 to 8 storms during the study period. The average storm intensity was 27.65 m/s (±3.45 m/s) of sustained winds, and the average intensity of the most-severe storm in each ZCTA was 33.55 m/s (±8.07 m/s).

### Summary statistics

There were 2,850,504 cardiovascular disease hospitalizations among pay-per-service Medicare beneficiaries in included ZIP codes in Florida in 1999–2016, and 756,028 respiratory disease hospitalizations. The demographic breakdown of these hospitalizations is shown in Table 1. The majority of those hospitalized were white people (87.9% of CVD hospitalizations and 87.7% of respiratory hospitalizations). More CVD and respiratory hospitalizations were for individuals 75–84 years of age (42.0% and 41.1%, respectively) compared to individuals 65–74 years of age (35.6% and 33.9% of hospitalizations) and those over 85 years (22.4% and 25.0% of hospitalizations). More women were hospitalized than men for both CVD (50.4%) and RD (55.9%). Supplementary Table 1 shows summary data for neighborhood-level characteristics at the ZCTA level. We found a strong correlation (≥0.85) for neighborhood-level characteristics within ZCTAs over time, and found that for any given variable, more than 92% of ZCTAs remained consistently above or below the median across the entire study period.

**Table 1:** Summary statistics for Medicare hospitalizations for included ZIPS (*n* = 796) in Florida, 1999–2016

Individual-level variable	Cardiovascular disease (CVD) hospitalizations		Respiratory disease (RD) hospitalizations	
	*n* (hospitalizations 1999–2016)	%	*n* (hospitalizations 1999–2016)	%
Race				
White	2,504,238	87.9%	662,900	87.7%
Black	201,230	7.1%	45,887	6.1%
Other	138,230	4.8%	45,455	6.0%
Missing	6806	0.2%	1786	0.2%
Age (years)				
65–74	1,013,761	35.6%	256,464	33.9%
75–84	1,198,372	42.0%	310,379	41.1%
85+	638,371	22.4%	189,185	25.0%
Sex				
Male	1,412,727	49.6%	333,322	44.1%
Female	1,437,777	50.4%	422,706	55.9%
Dual medicaid eligibility				
Yes	530,434	18.6%	206,162	27.3%
No	2,320,070	81.4%	549,866	72.7%

Next, we categorized ZCTAs based on whether they fell above or below the FL median value for each community-level variable (e.g., whether the average income of older adult residents in a given ZCTA was above (*n* = 398) or below (*n* = 398) the median value for all ZCTAs in FL) and compared TC metrics across these strata (Table 2). Significantly more TCs were observed in ZCTAs with above median housing value, income, education, percent of the population residing in urban areas, non-English speakers, and urban density, compared to those with below-median values for these characteristics. Storms were significantly more intense, based on sustained windspeed in these same communities. Despite the fact that they did not see more frequent storms, communities with above-median household density and those with below-median percent owner-occupied units also experienced significantly more severe storms on average. The intensity of the most extreme storm in each ZCTA was significantly higher in communities with above median housing value, income, education, non-English speakers, urban density, and percent urban than in those ZCTAs with below median values. The correlation matrix for all variables at the ZCTA level is shown in Supplementary Fig. 1.

**Table 2:** Tropical cyclone exposure metrics across included ZCTAs in Florida during the study period (1999–2016), stratified by whether they are above or below the Florida median

Variable	Average number of tropical cyclones	Average storm intensity (sustained windspeed, m/s)	Average intensity of most extreme storm (sustained windspeed, m/s)
Household density (residents/household)			
Above median	4.24 (1.49)	27.83 (3.39)	34.03 (7.56)
Below median	4.10 (1.59)	27.27 (3.36)	33.6 (8.67)
*p* value	0.176	**0.019**	0.452
Owner-occupied units (%)			
Above median	4.09 (1.54)	27.07 (3.42)	33.37 (8.87)
Below median	4.25 (1.55)	28.04 (3.28)	34.26 (7.3)
*p* value	0.148	**<0.001**	0.123
Housing value (USD)			
Above median	4.56 (1.42)	28.11 (2.83)	35.60 (7.72)
Below median	3.78 (1.56)	26.99 (3.78)	32.03 (8.16)
*p* value	**<0.001**	**<0.001**	**<0.001**
Percent below the poverty line (%)			
Above median	4.09 (1.55)	27.7 (3.41)	33.39 (7.54)
Below median	4.25 (1.53)	27.4 (3.36)	34.25 (8.67)
*p* value	0.136	0.202	0.136
Household income (USD)			
Above median	4.40 (1.54)	27.83 (3.36)	35.24 (8.41)
Below median	3.94 (1.52)	27.28 (3.39)	32.39 (7.6)
*p* value	**<0.001**	**0.022**	**<0.001**
Bachelor’s degree (%)			
Above median	4.46 (1.57)	27.79 (3.17)	34.92 (8.16)
Below median	3.88 (1.46)	27.31 (3.58)	32.71 (7.97)
*p* value	**<0.001**	**0.047**	**<0.001**
Non-english speakers (%)			
Above median	4.60 (1.39)	28.34 (2.74)	35.25 (7.04)
Below median	3.74 (1.57)	26.77 (3.77)	32.39 (8.88)
*p* value	**<0.001**	**<0.001**	**<0.001**
Population density (population/mi2)			
Above median	4.52 (1.5)	28.35 (3.13)	35.30 (7.51)
Below median	3.77 (1.5)	26.63 (3.44)	32.12 (8.49)
*p* value	**<0.001**	**<0.001**	**<0.001**
Percent of population living in urban areas (%)			
Above median	4.60 (1.52)	28.5 (3.14)	35.57 (7.51)
Below median	3.74 (1.44)	26.6 (3.36)	32.06 (8.36)
*p* value	**<0.001**	**<0.001**	**<0.001**
Percent black residents (%)			
Above median	4.08 (1.73)	27.78 (3.74)	33.62 (7.77)
Below median	4.26 (1.33)	27.32 (2.97)	34.02 (8.49)
p value	0.094	0.052	0.489

*p* values are for two-sided *t* tests, *α* = 0.05.

Bolded values show significance at *α* = 0.05.

### Statistical analyses

**Fig. 2:**
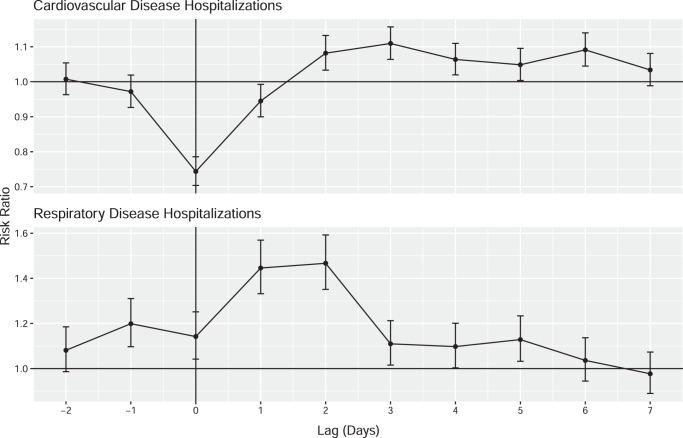
Overall association between tropical cyclone exposure and CVD and RD hospitalization risk in Florida ZCTAs (*n* = 796) from 1999 to 2016, from 2 days before to 7 days after a storm day (day 0). Dots show the point estimates and error bars represent 95% confidence intervals. Source data provided as a Source Data file.

In our primary analysis, we assessed the overall association between TC exposure and CVD and RD hospitalization risk (Fig. 2). We found that the risk of cardiovascular disease hospitalization decreased significantly on the day of TC exposure (RR: 0.74, 95% CI: 0.70, 0.79), and the day following a storm (RR: 0.94, 95% CI: 0.90, 0.99), and then was elevated for 3–6 days after storm exposure. We did not observe a statistically significant change in the risk of CVD hospitalizations over the whole 10-day TC period (2 days before the day of the storm, and 7 days following the storm) (RR: 1.04, 95% CI: 0.87, 1.24). In contrast, the risk between TC exposure and RD hospitalization risk showed that hospitalizations increased the day before TC exposure (RR: 1.20, 95% CI: 1.10, 1.31) and remained elevated for 5 days after the storm. The risk of RD hospitalizations increased significantly over the 10-day period (RR: 4.37, 95% CI: 3.08, 6.19).

**Fig. 3:**
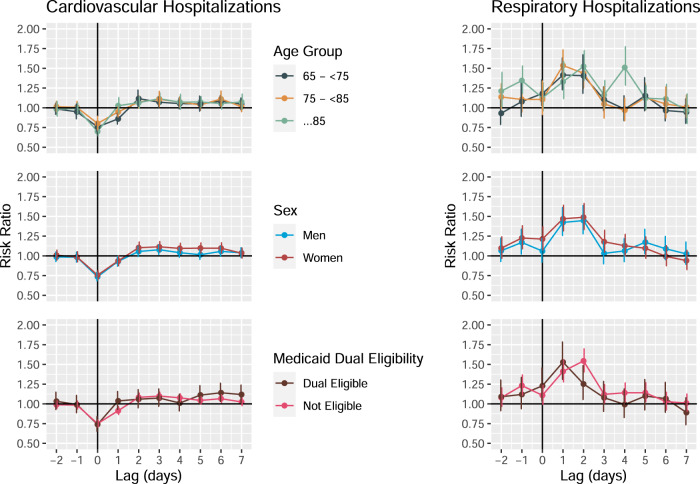
Association between tropical cyclone exposure and CVD and RD hospitalization risk in Florida ZCTAs (*n* = 796) from 1999 to 2016, from 2 days before to 7 days after a storm day, stratified by individual-level characteristics (age group, sex, and Medicaid dual eligibility). Dots show the point estimates and error bars represent 95% confidence intervals. Source data provided as a Source Data file.

Last, we stratified our models to assess whether hospitalization risk differs by age category, sex, and neighborhood characteristics. We found limited evidence for effect modification by age group, sex, or Medicaid dual-eligibility for CVD or RD (Fig. 3, full results Supplementary Table 2). We were unable to assess effect modification by race because ~90% of those hospitalized were white people.

**Fig. 4:**
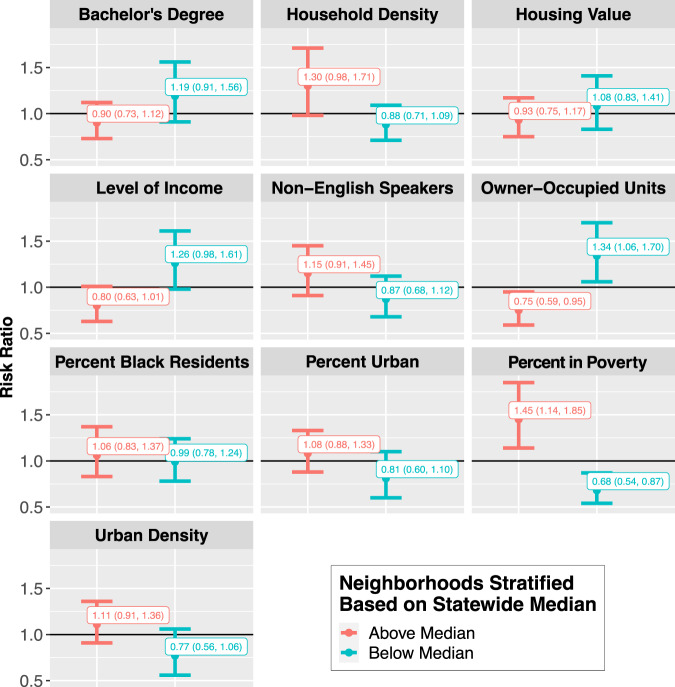
Cumulative association between tropical cyclone exposure and CVD hospitalization risk in Florida ZCTAs (*n* = 796) from 1999 to 2016, by lag (from 2 days before to 7 days after a storm day), stratified by neighborhood-level characteristics (above vs. below median). Dots show the point estimates and error bars represent 95% confidence intervals. Source data provided as a Source Data file.

However, the association between TCs and hospitalizations did appear to differ by neighborhood-level characteristics for both cardiovascular and respiratory disease. Cumulatively, the risk of CVD hospitalizations (Fig. 4) during 10-day TC exposed periods compared to matched unexposed periods differed for ZCTAs with higher levels of poverty (RR: 1.45, 95% CI: 1.14, 1.85) and those with lower poverty (RR: 0.68, 95% CI: 0.54, 0.87). In ZCTAs with lower percent owner-occupied units, there was an increased risk of CVD hospitalization during 10-day storm periods compared to non-storm periods (RR: 1.34, 95% CI: 1.06, 1.70), but in ZCTAs with higher percent owner-occupied units, there was a decreased risk of CVD hospitalization during 10-day storm periods compared to non-storm periods (RR: 0.75; 95% CI: 0.59, 0.95). Similar but non-significant patterns were observed based on whether ZCTAs were above or below the median for education, percent non-English speakers, percent urban, population density, household density, income, and housing value; the point estimates show an increased risk of hospitalization in communities that are less educated, more urban, lower income, and have more non-English speakers.

**Fig. 5:**
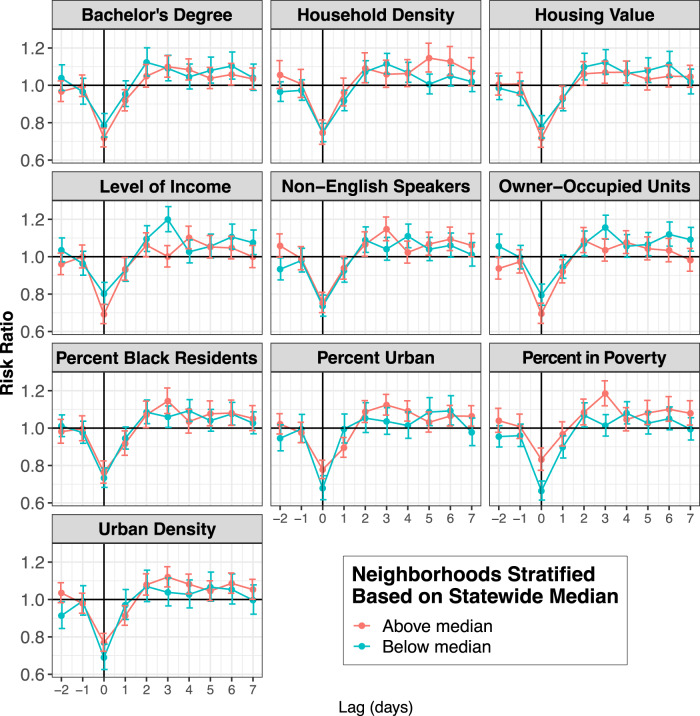
Association between tropical cyclone exposure and CVD hospitalization risk in Florida ZCTAs (*n* = 796) from 1999 to 2016, by lag (from 2 days before to 7 days after a storm day), stratified by neighborhood-level characteristics (above vs. below median). Dots show the point estimates and error bars represent 95% confidence intervals. Source data provided as a Source Data file.

Figure 5 shows the association between TCs and CVD hospitalization risks by daily lag (full results in Supplementary Table 3). In the unstratified analysis, on the closest storm day (lag 0) the risk of cardiovascular disease hospitalization was significantly lower than on non-TC days. However, when stratifying by level of poverty, the risk of CVD hospitalization on the closest storm day (lag 0) was significantly lower in lower-poverty ZCTAs compared to in higher-poverty ZCTAs. Further, on lag day 3, the association was significantly higher in higher-poverty communities than in lower-poverty communities.

**Fig. 6:**
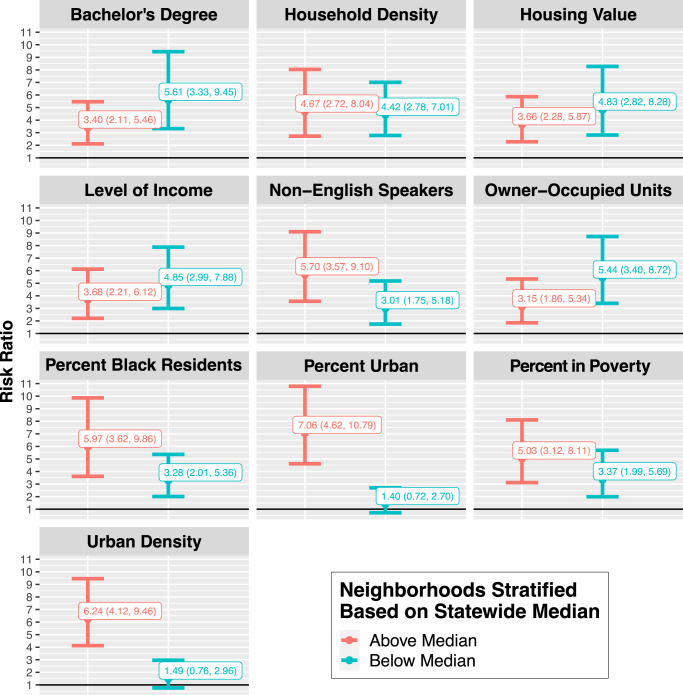
Cumulative association between tropical cyclone exposure and RD hospitalization risk in Florida ZCTAs (*n* = 796) from 1999 to 2016, from 2 days before to 7 days after a storm day, stratified by neighborhood-level characteristics (above vs. below median). Dots show the point estimates and error bars represent 95% confidence intervals. Source data provided as a Source Data file.

The cumulative risk of RD hospitalizations over the 10-day period (Fig. 6) also varied, but was based on different neighborhood-level characteristics. ZCTAs with above median density or above median percent urban had increased risk of RD hospitalization over the 10-day period (respectively, RR: 6.24, 95% CI: 4.12, 9.46, and RR: 7.06, 95% CI: 4.62, 10.79), but areas with below median density or percent urban did not have significant increases in risk.

**Fig. 7:**
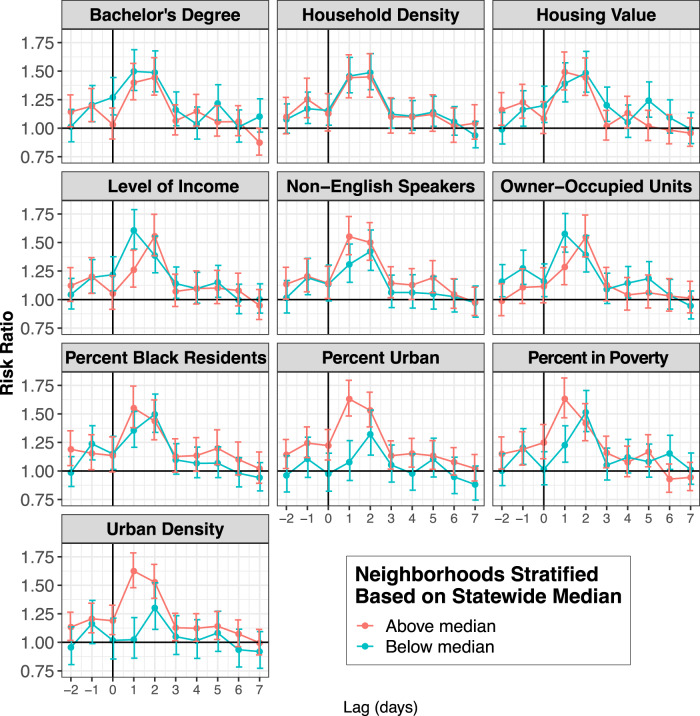
Association between tropical cyclone exposure and RD hospitalization risk in Florida ZCTAs (*n* = 796) from 1999 to 2016, from 2 days before to 7 days after a storm day, stratified by neighborhood-level characteristics (above vs. below median). Dots show the point estimates and error bars represent 95% confidence intervals. Source data provided as a Source Data file.

Figure 7 shows daily lags for RD hospitalization risk comparing the 10-day exposed TC period to unexposed periods (full results show shown in Supplementary Table 4). On the day of TC exposure (lag 0), we observed an increased risk of RD hospitalizations in ZCTAs that had below median education, percent Black people in the population, household density, owner-occupied units, housing value, and income. However, in ZCTAs above the FL median for these variables, there was no significant difference between RD hospitalizations on TC days compared to unexposed days. Further, in ZCTAs that were above the FL median for poverty, percent non-English speakers, urban density, and percent urban, there was an increased risk of RD hospitalizations on storm days compared to unexposed days, while in ZCTAs below the FL median for these variables there was no significant change in RD hospitalizations on TC days.

## Discussion

This study of over 3.5 million Medicare hospitalizations in the state of Florida from 1999 to 2016 provides suggestive evidence that the association between TCs and CVD and RD hospitalizations varies based on neighborhood-level characteristics, specifically poverty and homeownership (CVD) and urbanicity (RD). It is important to underscore that these neighborhood-level features are not in and of themselves the causes of the observed increase in risk. Instead, they are proxies for a wide array of other factors that could be affecting risk. More research is needed to understand the mechanisms underlying the associations presented in this paper; however, in the following subsections, we discuss a number of possible theories that could begin to explain these results.

In contrast to our findings at the neighborhood level, we did not find evidence that this association varies based on individual-level characteristics (age, sex, Medicaid dual eligibility). These findings contrast with other environmental exposures, such as air pollution^16^ and temperature^17^. Future studies should investigate wider age ranges (as we focused only on older adults), as well as other proxies for socioeconomic status besides Medicaid dual eligibility (such as level of income or level of education), and other individual-level characteristics that have been associated with health disparities following single storms, including race^13,18^ or whether a person has health insurance^19^ or pre-existing chronic health conditions^20^. Notably, over 80% of those hospitalized during the study period were white people. These results are relatively consistent with other studies that use Medicare data in Florida^21^, and may reflect that historically the proportion of white people has been higher among older adults in America than across the general population^22^. However, future research should focus on non-white racial groups, as well as on recent and undocumented immigrants, the latter of whom are not captured in this dataset but may be a potentially high-risk subpopulation. Overall, this research contributes to a significant gap in the literature on the health disparities associated with TCs.

### Cardiovascular disease

The overall risk of cardiovascular disease did not change significantly over the 10-day TC exposure period compared to unexposed periods. We did, however, observe a significant decrease in CVD hospitalizations on the TC-exposed day and the day following the storm. This could reflect that conditions may make it unsafe to travel, leading to delays in seeking care or reductions in the availability of emergency services^23,24^. These findings are consistent with other research focusing on multiple TCs across the United States^11^. However, when stratifying by neighborhood-level features, we found an increased risk over the 10-day TC exposure period in ZCTAs with above-median levels of poverty (i.e., neighborhoods with higher levels of poverty) or below median percentages of owner-occupied units (i.e., neighborhoods with more renters and fewer homeowners).

Two important mechanisms that have been proposed between TCs and CVD hospitalizations include the impacts of psychosocial and post-traumatic stress associated with storm exposure^5,25^ and disruption to healthcare services^26^. Individuals residing in communities with higher levels of poverty may experience higher storm-related stress associated with exposure^27^. Psychological distress among storm survivors has been associated with storm-related damage (which can be caused by high winds or flooding) and the experience of storm-related stressors such as lacking fresh water or food, or not knowing about the safety of a loved one^27^. In addition to the psychosocial impacts of the storm, the baseline health status of the community may impact the association between TCs and hospitalizations: individuals in communities with higher levels of poverty may have increased cardiovascular disease risk factors^28^. TCs may disrupt access to medication or interfere with the ability to receive medical treatment, which may be particularly significant for individuals with pre-existing conditions. Combined, these factors could contribute to an increased risk of cardiovascular disease hospitalizations associated with TC wind exposure in communities with higher levels of poverty. We also observed consistent but non-significant patterns across the other community-level variables, with point estimates showing increased risk of hospitalization in communities that are less educated, more urban, lower income, and have more non-English speakers, supporting the possibility that neighborhood-level deprivation may be associated with increased risk of adverse health outcomes associated with TCs.

Despite the increased risk of cardiovascular hospitalization associated with storm exposure in communities with higher levels of poverty, we did not find that these communities experienced more frequent or severe storms than communities with less poverty. This may reflect the concentration of wealthy communities along the Florida coastline^29^. However, these findings highlight the important difference between physical vulnerability (i.e., whether a community is likely to be exposed to storms) and social vulnerability *(*i.e., whether a community is able to respond to and recover from storm exposures)^30–32^. Specifically, this indicates that the elevated health risk in these communities that we observed in this study is not simply attributable to higher levels of exposure (as these communities are not experiencing more frequent or stronger storms) but instead likely results from other vulnerabilities or systemic inequities in communities’ abilities to cope with^25^ or recover from TC exposure^33^. For example, communities with higher levels of poverty may have more low-cost, affordable housing which may be particularly susceptible to damage associated with TC winds or flooding^34^, thus exacerbating potential health impacts of storm exposure.

We also observed a significant reduction in cardiovascular hospitalizations during TC exposure periods among communities with below-median levels of poverty or above median levels of owner-occupied units. Further, on the day of TC exposure, individuals in communities with lower levels of poverty experienced a significantly lower risk of CVD hospitalization than those in communities with higher levels of poverty. These neighborhood-level differences could be associated with different types of cardiovascular hospitalizations in different communities. Previous research has found increases in acute myocardial infarction hospitalizations associated with TCs, but decreases in non-acute hospitalizations^11^. Future research should investigate whether disaggregated cardiovascular hospitalizations vary across neighborhood-level sociodemographic characteristics.

Alternatively, the reduction in CVD hospitalizations in communities with lower poverty and home ownership may reflect differing evacuation rates. Overall evacuation in Florida can be quite high (for example, in the 2004 hurricane season, an estimated one-quarter of Florida’s population evacuated prior to at least one hurricane^35^), but data on evacuation rates are sparse and usually limited to specific storms and specific counties. There is some research to indicate that higher individual-level socioeconomic status may be associated with increased rates of evacuation^35^; however, there is limited research assessing whether evacuation rates vary by neighborhood-level sociodemographic characteristics. The link between homeowners and evacuation behavior is even less clear; in many cases, homeowners are less likely to evacuate than renters^35^, but this varies widely depending on other factors related to social capital and cohesion^36^. Future research should attempt to incorporate evacuation behavior into assessments of TC-related health impacts to understand whether differential evacuation behavior could be driving the observed community-level health disparities. This work should also be attentive to changes in early-warning systems, which have improved over time^37^.

### Respiratory disease

We found an increased risk of respiratory disease hospitalizations during TC periods, which is consistent with existing literature^3,11^. When stratifying by neighborhood-level features we found that more urban areas (based on percent urban or population density) had an elevated risk of RD hospitalizations during TC periods while less urban areas experienced no significant increase in RD hospitalizations. Further, more urban communities had a significant increase in RD hospitalizations beginning the day before storm exposure and lasting until 5 days after exposure. Less urban communities only had significant increases in hospitalizations 2 days after the storm.

There are a number of possible causal mechanisms through which the risk of RD hospitalization associated with TCs might be higher in more urban communities. Disparities may reflect the pre-existing health status of the population, as asthma and allergies among adults tend to be more prevalent in urban than in rural areas^38^. These respiratory illnesses may be exacerbated by TCs, either through stress and psychological impacts of storm exposure^7^ or through exposure to mold (which can start to form in as little as 24 hours after flooding)^39^. Further, rising air pollution in the days prior to thunderstorm events has been identified as one of the primary mechanisms of respiratory emergency department visits^40^. While this causal pathway is less well studied for TCs, it could be a plausible mechanism through which individuals in more urban neighborhoods might experience a heightened risk of respiratory hospitalizations during storm exposure periods^40^.

Finally, TCs may increase RD hospitalization via power outages which can disrupt access to ventilators and oxygen (which may be particularly significant for older adults)^41^. However, research on hurricane-related power outages in Florida found that outages were often more widespread and long-lasting in rural areas than in urban areas^42^. Given that our results show significant increases in more urban neighborhoods but not in less urban neighborhoods, power outages may not be the primary causal mechanism driving the observed health disparities.

We did not find significant variation across individual-level characteristics. However, when examining differences across age groups, the risk between TC and RD is only statistically significant in the ≥85 years age group for the 2 days prior to the storm. This could indicate that there is an increased risk among older adults for RD, which might become more apparent with a larger sample size. These individuals may have greater pre-existing illnesses, making them potentially more susceptible to the negative effects of tropical cyclones. Future research should investigate the potential for individual-level disparities, especially for RD hospitalizations, across a broader age range.

### Limitations

This study has limitations. First, exposure misclassification is possible. We downscaled county-level windspeed data to ZCTA-level, using an area-weighted average for ZCTAs that crossed multiple counties. However, TCs are significantly larger than ZCTAs (and counties)^43^. Any misclassification is likely non-differential as we do not expect that the misclassification would be associated with either respiratory or cardiovascular hospitalizations. Thus, any resulting bias would likely be toward the null. Second, we used data from the U.S. Census at the ZCTA level to assign neighborhood-level features. ZIP code-level data are commonly used to assess the neighborhood-level health disparities associated with environmental exposures^44^. However, administrative boundaries are not necessarily representative of communities and may include substantial sociodemographic heterogeneity. Third, we were unable to assess the impact of evacuation and displacement, which may play a significant role in driving health risks associated with tropical cyclones. More research is needed to understand how evacuation behavior varies by neighborhood in Florida, and how this affects the health impacts of TCs. Fourth, this study does not take into account the impact of multiple tropical cyclones within a single season. Future research should explicitly investigate the role of repeated and cumulative exposures across single hurricane seasons. Fifth, our focus on Medicare beneficiaries limits the generalizability of our findings to older populations in Florida. Future research investigates health disparities associated with TCs in larger populations and outside the state of Florida. Finally, the neighborhood-level composition may change over time. In this paper, we created fixed estimates for each community-level variable for each ZCTA over the study period and categorized ZCTAs based on whether they fell above or below the median value for each characteristic. While most ZCTAs stayed consistent over time (above or below the median), our approach does not allow for variation in neighborhood-level features over time. Future research should address this temporal aspect, and should consider a finer categorization of neighborhood-level features.

Overall, these findings contribute to a growing body of literature on the health impacts of TCs. Our project focused specifically on health disparities associated with TC exposure, which remains an understudied but critical topic. We found suggestive evidence that the health effects of TCs vary across neighborhood-level characteristics, and are elevated in communities with more poverty and less homeownership (for CVD hospitalizations) and in denser, more urban communities (for RD hospitalizations). Building on these results, future research should assess possible causal mechanisms that might be driving the observed disparities in the health impacts of TCs, including the potential for differential evacuation patterns, the impact of power outages, and access to hospitals. A better scientific understanding of TC-associated health disparities will help governments and policymakers improve plans for disaster preparedness and response that are specifically targeted to address the needs of high-risk populations.

## Methods

This project complies with all relevant ethical regulations and was approved by the Institutional Review Board at Yale University. The use of hospitalization and census records comply with the terms and conditions of the original databases.

### Tropical cyclone exposure

We obtained county-level TC exposure from the “hurricaneexposure” package in R^45^. This package is described in detail elsewhere^43^, but in brief, was developed using data on Atlantic-basin storms from the National Hurricane Center’s revised hurricane database (HURDAT2) for all storms that came within 250 km of at least one US county. For each storm, the ground-level peak sustained wind speed was modeled at each county’s population mean center using a double exponential wind speed model^43^. The “hurricaneexposure” package has increasingly been utilized in research assessing the impacts of TCs across the US^3,11,46–48^.

For this study, we considered a county to be exposed if it experienced local storm-associated winds of ≥21 m/s. Epidemiological studies have used a variety of metrics to operationalize TC exposure, including rainfall, flooding, damage, and individual-level stressors or perceived impact^49^. However, there is no consensus on which measure is most appropriate^50^. In this study, we used modeled data on all TCs (with sustained windspeeds ≥21 m/s, corresponding to strong gale-force winds on the Beaufort Scale) in Florida during the study period. These storms would be considered tropical storms or higher on the Saffir Simpson scale. We selected windspeed as the exposure measure because it is a commonly used metric for classifying storm severity^51^ and it has been used elsewhere to assess the health impacts of tropical cyclones^3,11,12,50^. In using windspeed as a proxy for exposure, we assume that during storms with gale-force winds (or higher) other important threats may be present as well (including high rainfall or flooding) which may be more causally linked to health outcomes.

To assess effect modification by community-level features we downscaled our exposure data to the ZCTA level. Each ZCTA was assigned the TC exposures of the county in which it was located. ZCTAs located within multiple counties (i.e., crossing county borders) were assigned the TC exposures of the county in which the highest percentage of the ZCTA (by land area) was located. We calculated summary statistics for TC metrics: the number of TCs, average intensity based on sustained windspeed, and average intensity of the most extreme storm for each individual ZCTA.

### Hospitalization records

We obtained daily hospitalization data based on the residential ZIP code of the patient for all pay-per-service Medicare beneficiaries (≥65 years) in Florida (1999–2016) for cardiovascular disease ([CVD]: International Classification of Diseases [ICD]−9, 390–459; ICD-10, I00–I99) and respiratory disease ([RD]: ICD-9, 464–466, 480–487, and 490–493; ICD-10, J06.9; J20.9; J18.9; J43.9; J40; J41.0; J41.1; J41.8; J42; J44.0; J44.1; J44.9; J47.1; J47.9; J67.0-J67.9). ZIP codes reflect participants’ primary residential addresses. This data does not include Medicare Advantage (Part C) enrollees, for whom claims are processed separately^52^. However, traditional Medicare enrollees (included here) and Medicare Advantage enrollees (not included here) do not differ significantly by age, income, race, or chronic conditions^53^. We aggregated daily hospitalizations from ZIP to ZCTA for analysis. For all Medicare records we obtained individual-level characteristics including age (65 to 74 years, 75 to 84 years, ≥85 years), sex (male, female), race (Black, white, other), and dual Medicaid eligibility (yes, no), which serves as an indicator of low socioeconomic status. Medicare data is highly representative of older adults in American, as 98% of Americans aged 65 years or older are insured by Medicare^52^. However, our study does not capture morbidities that did not result in hospitalization.

### Census data

To explore neighborhood characteristics, we used data from the U.S. Decennial Census and the American Community Survey (ACS). We considered education (percent of older adults in the ZCTA with a bachelor’s degree), income (percent of older adults living in poverty; the average income of older adults in the ZCTA, including retirement benefits and other sources of income), primary language (percent of older adults who speak English “not well” or “not at all”), housing (household density; median housing unit value), urbanicity (population density; percent urban), and racial composition (percent Black people in the population). We used an area-weighted average to interpolate from 2000 ZCTA boundaries to 2010 ZCTA boundaries, a common approach to account for changes in Census boundaries over time^54^. We used time-weighted averaging to assign values of each variable for each ZCTA across the study period. ZCTAs were then categorized based on whether they fell above or below the median value for each community-level variable (e.g., whether the average income of older adults in a given ZCTA-level was above or below the median value for all ZCTAs in FL) across the study period. We chose to use a coarse categorization of census variables (above or below the median) because we assumed that most ZCTAs would not move across this threshold during the study period. To test this assumption, we calculated Pearson’s correlation between the beginning and the end of the study period for each variable within each ZCTA. We also calculated the percentage of ZCTAs that failed to meet this assumption (i.e., crossed the median threshold over the course of the study period). We then summarized census variables (mean, median, standard deviation) for all ZCTAs. Lastly, we calculated the correlation matrix for all ten variables using Pearson’s correlation coefficient.

### Statistical analyses

We restricted our analysis to ZCTAs with complete data (*n* = 794, or 80.8% of the 983 ZCTAs in Florida), and those with at least one tropical cyclone exposure during the study period. We excluded ZCTAs that were uninhabited areas (i.e., bodies of water, parks, etc.). Next, we stratified Medicare beneficiaries based on whether the ZCTA in which they resided fell above or below the FL median value for each community-level variable. We compared TC metrics (the number of cyclones, average intensity based on sustained windspeed, and average intensity of most extreme storm) across these strata.

To assess the association between TC exposure and cause-specific hospitalization risk, we matched the day of storm’s closest approach (referred to as “TC-exposed day” throughout this paper) within each ZCTA to 10 “unexposed-days” from different years, an approach based on previous work^3^. These unexposed-days were randomly selected days that fell within a seven-day window of the day-of-year of the TC (to account for seasonality). We did not allow days that fell within a 3-day period of a different TC in the same ZCTA to be selected as controls. We obtained daily hospitalization records for the 10-day period around each TC-exposed day (2 days before and seven days after) and each matched non-exposed day (resulting in 110 days for each TC in each zip code). This matching procedure was developed based on similar matched designs used in environmental epidemiology studies^3,55,56^. A visualization of the matching process is shown in Supplementary Fig. 2. We then used a mixed effect Poisson regression model to estimate the relative risk (RR) of hospitalizations for TC-periods (from 2 days before to 7 days after) compared to matched non-TC-periods, incorporating a distributed lag for storm exposures:

1 $${{\log }}\left[E\left({Y}_{t}^{Z}\right)\right]=\,\alpha+{\alpha }^{{{{{{\rm{Z}}}}}}}+\mathop{\sum}\limits_{l=-2}^{7}{\beta }_{l}{x}_{t+l,z}^{Z}+{{\log }}\left({n}_{T}^{Z}\right)+{{{{{{\boldsymbol{\delta }}}}}}^{\prime}{{{{{\rm{DOW}}}}}}}_{t}+{{{{{{\boldsymbol{\gamma }}}}}}^{\prime}{{{{{\rm{Year}}}}}}}_{t}$$

where ${Y}_{t}^{Z}$ is the total count of cause-specific hospital admissions in ZCTA *z* on day *t;* $\alpha$ is a fixed intercept; ${\alpha }^{Z}$ is a random intercept for ZCTA; ${\sum }_{l=-2}^{7}{\beta }_{l}{x}_{t+l}^{Z}$ is a distributed lag function of storm exposure variable $x$, ${\beta }_{l}$ is the association between TC exposure and hospitalization on lag *l* on day $t$, and ${x}_{t+l,z}^{Z}$ is an indicator that represents whether day $t$ on lag *l* in ZCTA z is part of a TC-exposed day (or is part of a matched unexposed period)*;* ${{\log }}\left({n}_{T}^{Z}\right)$ is an offset for the number of Medicare beneficiaries; $\delta$ is a vector of coefficients for day of week; and $\gamma$ is a vector of coefficients for year.

We first quantified the overall associations between TC exposure and cause-specific hospitalization risk. We next stratified our analyses to investigate whether TC-related hospitalization risk differs by individual and neighborhood characteristics.

Analyses were conducted using the R Statistical Software, version 3.6.3. Distributed lag nonlinear models were specified through the “dlnm” package^57^.

### Reporting summary

Further information on research design is available in the Nature Portfolio Reporting Summary linked to this article.

## Supplementary information

Supplementary information

Reporting Summary

## Data Availability

Exposure data are available through the “hurricaneexposure” package in R (https://cran.r-project.org/web/packages/hurricaneexposure/vignettes/hurricaneexposure.html). These data are based on tropical cyclones recorded in the HURDAT2 dataset (https://www.aoml.noaa.gov/hrd/hurdat/Data_Storm.html). Health data (Medicare enrollees dynamic cohort) is available upon purchase and after an application process, from the Centers for Medicare & Medicaid Services (https://www.cms.gov/research-statistics-data-and-systems/cms-information-technology/accesstodataapplication). Source data are provided with this paper.

## Source data

Source Data

## Code availability

Code used in this project is available online at (https://github.com/kateburrows/tropical_cyclone_NatureComm)^58^.
