# Supplementary material for: Health disparities among older adults following tropical cyclone exposure in Florida
Source: Nat Commun. 2023 Apr 19;14:2221. doi: 10.1038/s41467-023-37675-7 (PMC10115860; doi:10.1038/s41467-023-37675-7)

Supplementary Information

Health Disparities Among Older Adults following Tropical Cyclone Exposure in Florida
K. Burrows; G.B. Anderson; M. Yan; A. Wilson; M.B. Sabath; J.Y. Son; H. Kim; F. Dominici; M.L. Bell

Supplementary Table 1. Neighborhood-level characteristics from the United States Decennial Census and American Community Survey (ACS) for ZCTAs with complete data (n=796) for the study period (1999-2016).

Supplementary Table 2. Association between tropical cyclone exposure and cardiovascular and respiratory disease hospitalization risk in Florida ZCTAs (n=796) from 1999-2016, from two days before to 7 days after a storm day, stratified by individual-level characteristics (age group, sex, and Medicaid dual eligibility).

Supplementary Table 3. Association between tropical cyclone exposure and cardiovascular hospitalization risk in Florida ZCTAs (n=796) from 1999-2016, by lag (from two days before to 7 days after a storm day), stratified by neighborhood-level characteristics (above vs. below median).

Supplementary Table 4. Association between tropical cyclone exposure and respiratory hospitalization risk in Florida ZCTAs (n=796) from 1999-2016, by lag (from two days before to 7 days after a storm day), stratified by neighborhood-level characteristics (above vs. below median).

Supplementary Figure 1. Correlation matrix between neighborhood-level characteristics.

Supplementary Figure 2. Visualization of matching procedure.

Table S1. Neighborhood-level characteristics from the United States Decennial Census and American Community Survey (ACS) for ZCTAs with complete data (n=796) for the study period (1999-2016).

Grouping	Variable	Mean (sd)	Median	Autocorrelation[i]	ZCTAs with significant change (%)[ii]
Housing	Household density (residents/household)	2.45 (0.36)	2.44	0.87	0.06
	Owner occupied units (%)	72.30 (13.80)	75.65	0.90	0.07
	Housing Value (USD)	160,795 (96,298)	133,227	0.92	0.05
Income	Below the poverty line (%)	10.24 (6.25)	8.51	0.86	0.05
	Household income (USD)	38,118 (12,465)	38,118	0.86	0.07
Education	Bachelor’s degree (%)	21.47 (11.40)	18.79	0.86	0.07
Language	Non-English speakers (%)	6.79 (11.90)	2.31	0.95	0.06
Urbanicity	Population density (population/mi2)	1,971.74 (2,493.45)	1,070.20	0.99	0.06
	Population living in urban areas (%)	77.89 (32.37)	95.64	0.90	0.03
Racial Composition	Percent of Black residents (%)	13.28 (16.34)	7.71	0.95	0.06

[i] Average correlation between start and end of study period within each ZCTA for a given variable.
[ii] Percent of ZCTAs that were not consistently above or below the median value across the entire study period.

Table S2. Association between tropical cyclone exposure and cardiovascular and respiratory disease hospitalization risk in Florida ZCTAs (n=796) from 1999-2016, from two days before to 7 days after a storm day, stratified by individual-level characteristics (age group, sex, and Medicaid dual eligibility).

	Cardiovascular Disease							Respiratory Disease						
	Age Group			Sex		Medicaid Dual Eligibility		Age Group			Sex		Medicaid Dual Eligibility	
	65-<75	75 -<85	≥85	Men	Women	Not Eligible	Dual Eligible	65 -<75	75 -<85	≥ 85	Men	Women	Not Eligible	Dual Eligible
Lag -2	0.99 (0.92, 1.06)	1.01 (0.95, 1.08)	0.98 (0.89, 1.08)	0.99 (0.93, 1.05)	1.01 (0.95, 1.07)	0.99 (0.94, 1.04)	1.03 (0.93, 1.15)	0.93 (0.79, 1.1)	1.14 (0.99, 1.31)	1.21 (1.01, 1.45)	1.07 (0.93, 1.22)	1.1 (0.97, 1.24)	1.08 (0.97, 1.2)	1.09 (0.91, 1.3)
Lag -1	1.01 (0.93, 1.08)	0.99 (0.93, 1.06)	0.95 (0.86, 1.05)	0.98 (0.92, 1.05)	0.99 (0.93, 1.05)	0.98 (0.94, 1.03)	0.99 (0.89, 1.11)	1.11 (0.94, 1.29)	1.34 (1.18, 1.53)	1.08 (0.89, 1.31)	1.17 (1.02, 1.34)	1.23 (1.09, 1.38)	1.23 (1.11, 1.37)	1.12 (0.94, 1.33)
Lag 0	0.7 (0.64, 0.77)	0.76 (0.7, 0.82)	0.8 (0.72, 0.89)	0.74 (0.68, 0.79)	0.76 (0.7, 0.81)	0.75 (0.71, 0.79)	0.74 (0.65, 0.84)	1.13 (0.96, 1.32)	1.18 (1.03, 1.36)	1.11 (0.91, 1.34)	1.06 (0.92, 1.22)	1.21 (1.08, 1.37)	1.11 (0.99, 1.24)	1.23 (1.04, 1.45)
Lag 1	0.86 (0.79, 0.93)	0.95 (0.88, 1.02)	1.03 (0.93, 1.13)	0.93 (0.87, 0.99)	0.94 (0.88, 1)	0.91 (0.87, 0.96)	1.04 (0.93, 1.16)	1.42 (1.23, 1.63)	1.54 (1.36, 1.73)	1.33 (1.12, 1.59)	1.42 (1.26, 1.61)	1.47 (1.32, 1.64)	1.41 (1.28, 1.56)	1.53 (1.31, 1.78)
Lag 2	1.07 (1, 1.15)	1.07 (1, 1.14)	1.12 (1.02, 1.23)	1.06 (0.99, 1.12)	1.1 (1.04, 1.17)	1.08 (1.03, 1.14)	1.06 (0.95, 1.18)	1.44 (1.25, 1.66)	1.52 (1.35, 1.73)	1.41 (1.18, 1.67)	1.45 (1.28, 1.64)	1.49 (1.33, 1.66)	1.54 (1.41, 1.7)	1.25 (1.06, 1.49)
Lag 3	1.12 (1.04, 1.19)	1.07 (1.01, 1.14)	1.11 (1.01, 1.21)	1.08 (1.02, 1.14)	1.11 (1.05, 1.18)	1.1 (1.05, 1.15)	1.07 (0.97, 1.19)	1.16 (1, 1.35)	1.1 (0.96, 1.27)	1.05 (0.87, 1.27)	1.03 (0.9, 1.18)	1.18 (1.05, 1.32)	1.12 (1.01, 1.24)	1.08 (0.91, 1.28)
Lag 4	1.05 (0.99, 1.13)	1.07 (1.01, 1.14)	1.07 (0.98, 1.17)	1.04 (0.98, 1.1)	1.09 (1.03, 1.16)	1.08 (1.03, 1.13)	1.01 (0.91, 1.12)	0.98 (0.84, 1.15)	0.97 (0.84, 1.12)	1.51 (1.29, 1.77)	1.06 (0.93, 1.22)	1.13 (1, 1.27)	1.14 (1.03, 1.27)	0.99 (0.83, 1.19)
Lag 5	1.04 (0.97, 1.11)	1.07 (1.01, 1.14)	1.05 (0.96, 1.15)	1.01 (0.96, 1.08)	1.1 (1.04, 1.16)	1.04 (1, 1.09)	1.11 (1.01, 1.23)	1.13 (0.97, 1.31)	1.12 (0.98, 1.29)	1.15 (0.96, 1.38)	1.17 (1.03, 1.34)	1.1 (0.97, 1.24)	1.14 (1.03, 1.27)	1.1 (0.92, 1.31)
Lag 6	1.05 (0.98, 1.13)	1.08 (1.02, 1.16)	1.11 (1.02, 1.21)	1.06 (1, 1.12)	1.1 (1.03, 1.16)	1.07 (1.02, 1.12)	1.14 (1.03, 1.26)	1.11 (0.95, 1.3)	0.97 (0.83, 1.12)	1.05 (0.87, 1.26)	1.09 (0.95, 1.25)	0.99 (0.88, 1.13)	1.03 (0.92, 1.15)	1.07 (0.89, 1.27)
Lag 7	1.05 (0.98, 1.12)	1.01 (0.95, 1.08)	1.07 (0.98, 1.17)	1.04 (0.98, 1.1)	1.03 (0.97, 1.1)	1.02 (0.98, 1.07)	1.12 (1.01, 1.24)	0.95 (0.8, 1.11)	1.01 (0.88, 1.16)	0.97 (0.8, 1.18)	1.02 (0.89, 1.17)	0.94 (0.83, 1.07)	1.01 (0.91, 1.13)	0.89 (0.74, 1.08)

Note: 95% confidence intervals shown in parentheses.

Table S3. Association between tropical cyclone exposure and cardiovascular hospitalization risk in Florida ZCTAs (n=796) from 1999-2016, by lag (from two days before to 7 days after a storm day), stratified by neighborhood-level characteristics (above vs. below median).

	**Household density (residents/household)**		**Owner occupied units (%)**		**Housing value (USD)**		**Percent living below the poverty line (%)**		**Household income (USD)**	
	Below Median	*Above Median*	*Below Median*	*Above Median*	*Below Median*	*Above Median*	*Below Median*	*Above Median*	*Below Median*	*Above Median*
Lag -2	0.97 (0.91, 1.02)	1.06 (0.98, 1.13)	1.06 (1, 1.12)	0.94 (0.88, 1)	0.98 (0.92, 1.05)	1 (0.95, 1.06)	0.95 (0.9, 1.01)	1.04 (0.98, 1.11)	1.03 (0.97, 1.1)	0.96 (0.9, 1.02)
Lag -1	0.97 (0.92, 1.03)	1.01 (0.93, 1.09)	1 (0.93, 1.06)	0.97 (0.91, 1.04)	0.96 (0.89, 1.02)	1.01 (0.95, 1.07)	0.96 (0.9, 1.02)	1.01 (0.94, 1.08)	0.96 (0.9, 1.03)	1 (0.94, 1.06)
Lag 0	0.75 (0.7, 0.8)	0.75 (0.68, 0.81)	0.79 (0.74, 0.85)	0.7 (0.64, 0.75)	0.78 (0.72, 0.84)	0.72 (0.67, 0.77)	0.66 (0.61, 0.72)	0.83 (0.77, 0.89)	0.8 (0.74, 0.86)	0.69 (0.64, 0.75)
Lag 1	0.92 (0.86, 0.97)	0.96 (0.89, 1.04)	0.94 (0.88, 1.01)	0.92 (0.86, 0.98)	0.93 (0.86, 0.99)	0.93 (0.88, 1)	0.9 (0.84, 0.96)	0.97 (0.9, 1.03)	0.93 (0.87, 1)	0.93 (0.87, 1)
Lag 2	1.07 (1.01, 1.13)	1.09 (1.01, 1.17)	1.07 (1, 1.14)	1.09 (1.02, 1.16)	1.1 (1.03, 1.17)	1.06 (1, 1.13)	1.07 (1.01, 1.14)	1.08 (1.02, 1.15)	1.09 (1.03, 1.17)	1.06 (1, 1.13)
Lag 3	1.11 (1.06, 1.17)	1.06 (0.99, 1.13)	1.16 (1.09, 1.22)	1.03 (0.98, 1.1)	1.12 (1.06, 1.19)	1.07 (1.01, 1.13)	1.01 (0.96, 1.07)	1.18 (1.12, 1.25)	1.2 (1.13, 1.27)	1 (0.94, 1.06)
Lag 4	1.07 (1.02, 1.12)	1.06 (0.99, 1.14)	1.06 (1, 1.12)	1.08 (1.02, 1.14)	1.06 (1, 1.13)	1.07 (1.01, 1.13)	1.08 (1.02, 1.14)	1.05 (0.99, 1.11)	1.03 (0.97, 1.09)	1.1 (1.04, 1.16)
Lag 5	1.01 (0.95, 1.06)	1.15 (1.07, 1.23)	1.07 (1, 1.13)	1.04 (0.98, 1.11)	1.08 (1.02, 1.15)	1.03 (0.97, 1.09)	1.03 (0.97, 1.09)	1.08 (1.02, 1.15)	1.06 (0.99, 1.12)	1.05 (0.99, 1.11)
Lag 6	1.05 (1, 1.11)	1.13 (1.05, 1.21)	1.12 (1.06, 1.19)	1.03 (0.97, 1.1)	1.11 (1.04, 1.18)	1.05 (0.99, 1.11)	1.05 (0.99, 1.11)	1.1 (1.04, 1.17)	1.11 (1.04, 1.17)	1.05 (0.99, 1.11)
Lag 7	1.02 (0.97, 1.08)	1.07 (1, 1.15)	1.09 (1.03, 1.16)	0.98 (0.92, 1.04)	1.02 (0.95, 1.09)	1.05 (0.99, 1.11)	0.99 (0.94, 1.06)	1.08 (1.02, 1.15)	1.08 (1.01, 1.14)	1 (0.94, 1.06)

Note: 95% confidence intervals shown in parentheses.

Table S3. Continued

	Bachelor's degree (%)		Non-English speakers (%)		Population density (population/mi2)		Population living in urban areas (%)		Percent of Black residents (%)	
	Below Median	*Above Median*	*Below Median*	*Above Median*	*Below Median*	*Above Median*	*Below Median*	*Above Median*	*Below Median*	*Above Median*
Lag -2	1.04 (0.97, 1.11)	0.97 (0.91, 1.02)	0.93 (0.88, 0.99)	1.06 (1, 1.12)	0.91 (0.84, 0.99)	1.03 (0.98, 1.09)	0.95 (0.88, 1.02)	1.02 (0.97, 1.08)	1.01 (0.95, 1.07)	0.98 (0.92, 1.05)
Lag -1	0.97 (0.9, 1.04)	1 (0.94, 1.06)	0.98 (0.92, 1.05)	0.99 (0.93, 1.05)	0.99 (0.92, 1.07)	0.98 (0.93, 1.03)	1 (0.92, 1.07)	0.97 (0.92, 1.03)	0.98 (0.92, 1.04)	1 (0.93, 1.07)
Lag 0	0.78 (0.72, 0.85)	0.72 (0.67, 0.77)	0.74 (0.68, 0.79)	0.75 (0.7, 0.81)	0.69 (0.63, 0.76)	0.77 (0.72, 0.82)	0.68 (0.62, 0.75)	0.78 (0.73, 0.83)	0.73 (0.68, 0.79)	0.76 (0.71, 0.82)
Lag 1	0.95 (0.89, 1.02)	0.92 (0.86, 0.98)	0.92 (0.86, 0.99)	0.94 (0.88, 1)	0.97 (0.89, 1.05)	0.91 (0.86, 0.97)	0.99 (0.92, 1.08)	0.9 (0.84, 0.95)	0.95 (0.89, 1.01)	0.92 (0.85, 0.99)
Lag 2	1.12 (1.05, 1.2)	1.05 (0.99, 1.11)	1.09 (1.02, 1.16)	1.07 (1, 1.13)	1.07 (0.99, 1.16)	1.08 (1.02, 1.14)	1.05 (0.98, 1.14)	1.09 (1.03, 1.15)	1.09 (1.02, 1.15)	1.07 (1, 1.14)
Lag 3	1.09 (1.02, 1.16)	1.1 (1.04, 1.16)	1.04 (0.98, 1.1)	1.15 (1.09, 1.21)	1.04 (0.97, 1.12)	1.12 (1.07, 1.18)	1.04 (0.97, 1.11)	1.12 (1.07, 1.18)	1.06 (1, 1.12)	1.14 (1.08, 1.21)
Lag 4	1.05 (0.98, 1.11)	1.08 (1.03, 1.14)	1.11 (1.05, 1.17)	1.02 (0.97, 1.08)	1.03 (0.96, 1.1)	1.08 (1.03, 1.14)	1.01 (0.95, 1.09)	1.09 (1.04, 1.15)	1.09 (1.04, 1.15)	1.04 (0.97, 1.1)
Lag 5	1.08 (1.01, 1.15)	1.04 (0.98, 1.1)	1.04 (0.98, 1.1)	1.07 (1.01, 1.13)	1.07 (0.99, 1.15)	1.05 (0.99, 1.1)	1.09 (1.01, 1.16)	1.03 (0.98, 1.09)	1.04 (0.98, 1.1)	1.08 (1.01, 1.15)
Lag 6	1.1 (1.03, 1.18)	1.06 (1, 1.12)	1.06 (1, 1.13)	1.09 (1.03, 1.16)	1.05 (0.98, 1.14)	1.09 (1.03, 1.14)	1.09 (1.02, 1.17)	1.06 (1.01, 1.12)	1.08 (1.02, 1.14)	1.08 (1.01, 1.15)
Lag 7	1.04 (0.97, 1.11)	1.03 (0.98, 1.09)	1.01 (0.95, 1.08)	1.06 (1, 1.12)	1 (0.92, 1.08)	1.05 (1, 1.11)	0.98 (0.91, 1.05)	1.06 (1.01, 1.12)	1.03 (0.97, 1.09)	1.05 (0.99, 1.12)

Note: 95% confidence intervals shown in parentheses.

Table S4. Association between tropical cyclone exposure and respiratory hospitalization risk in Florida ZCTAs (n=796) from 1999-2016, by lag (from two days before to 7 days after a storm day), stratified by neighborhood-level characteristics (above vs. below median).

	**Household density (residents/household)**		**Owner occupied units (%)**		**Housing value (USD)**		**Percent living below the poverty line (%)**		**Household income (USD)**	
	Below Median	*Above Median*	*Below Median*	*Above Median*	*Below Median*	*Above Median*	*Below Median*	*Above Median*	*Below Median*	*Above Median*
Lag -2	1.08 (0.96, 1.21)	1.1 (0.95, 1.27)	1.16 (1.03, 1.31)	0.99 (0.86, 1.14)	0.99 (0.86, 1.14)	1.16 (1.03, 1.31)	1 (0.87, 1.15)	1.15 (1.02, 1.3)	1.04 (0.92, 1.19)	1.12 (0.98, 1.28)
Lag -1	1.17 (1.04, 1.32)	1.25 (1.09, 1.44)	1.27 (1.13, 1.43)	1.11 (0.97, 1.27)	1.16 (1.02, 1.33)	1.23 (1.09, 1.38)	1.2 (1.06, 1.37)	1.19 (1.05, 1.34)	1.19 (1.06, 1.35)	1.2 (1.05, 1.37)
Lag 0	1.16 (1.03, 1.30)	1.13 (0.98, 1.3)	1.16 (1.03, 1.31)	1.11 (0.97, 1.28)	1.20 (1.05, 1.37)	1.08 (0.95, 1.23)	1.01 (0.88, 1.17)	1.25 (1.11, 1.41)	1.22 (1.08, 1.38)	1.05 (0.92, 1.21)
Lag 1	1.46 (1.31, 1.62)	1.44 (1.27, 1.64)	1.58 (1.42, 1.75)	1.29 (1.13, 1.46)	1.39 (1.23, 1.57)	1.49 (1.34, 1.67)	1.23 (1.08, 1.4)	1.63 (1.47, 1.82)	1.61 (1.44, 1.79)	1.26 (1.11, 1.43)
Lag 2	1.49 (1.34, 1.65)	1.45 (1.27, 1.65)	1.39 (1.24, 1.56)	1.55 (1.37, 1.74)	1.48 (1.32, 1.67)	1.44 (1.29, 1.62)	1.51 (1.34, 1.71)	1.42 (1.27, 1.59)	1.39 (1.23, 1.56)	1.55 (1.38, 1.75)
Lag 3	1.12 (1, 1.26)	1.1 (0.96, 1.27)	1.09 (0.97, 1.23)	1.13 (0.99, 1.28)	1.2 (1.06, 1.36)	1.02 (0.9, 1.16)	1.05 (0.92, 1.2)	1.16 (1.03, 1.3)	1.14 (1.01, 1.29)	1.07 (0.94, 1.22)
Lag 4	1.1 (0.98, 1.24)	1.1 (0.95, 1.27)	1.14 (1.02, 1.29)	1.04 (0.91, 1.19)	1.05 (0.92, 1.2)	1.13 (1, 1.28)	1.12 (0.98, 1.28)	1.08 (0.95, 1.22)	1.1 (0.97, 1.24)	1.1 (0.96, 1.25)
Lag 5	1.14 (1.02, 1.28)	1.12 (0.97, 1.29)	1.18 (1.05, 1.33)	1.06 (0.93, 1.22)	1.24 (1.1, 1.41)	1.02 (0.89, 1.16)	1.08 (0.95, 1.24)	1.17 (1.04, 1.32)	1.15 (1.02, 1.3)	1.1 (0.96, 1.26)
Lag 6	1.06 (0.94, 1.19)	1.02 (0.88, 1.18)	1.04 (0.92, 1.18)	1.03 (0.9, 1.18)	1.09 (0.96, 1.25)	0.98 (0.86, 1.12)	1.15 (1.01, 1.31)	0.93 (0.81, 1.06)	1 (0.88, 1.13)	1.08 (0.94, 1.23)
Lag 7	0.94 (0.83, 1.06)	1.04 (0.9, 1.21)	0.94 (0.83, 1.07)	1.01 (0.88, 1.16)	0.99 (0.86, 1.14)	0.96 (0.84, 1.09)	1.01 (0.88, 1.16)	0.94 (0.83, 1.08)	1 (0.88, 1.14)	0.95 (0.83, 1.09)

Note: 95% confidence intervals shown in parentheses.

Table S4. Continued

	Bachelor's degree (%)		Non-English speakers (%)		Population density (population/mi2)		Population living in urban areas (%)		Percent of Black residents (%)	
	Below Median	*Above Median*	*Below Median*	*Above Median*	*Below Median*	*Above Median*	*Below Median*	*Above Median*	*Below Median*	*Above Median*
Lag -2	1.01 (0.88, 1.16)	1.14 (1.01, 1.29)	1.01 (0.88, 1.17)	1.14 (1, 1.28)	0.96 (0.8, 1.14)	1.13 (1.02, 1.26)	0.96 (0.82, 1.13)	1.14 (1.02, 1.28)	0.99 (0.86, 1.13)	1.19 (1.05, 1.35)
Lag -1	1.2 (1.06, 1.37)	1.19 (1.06, 1.35)	1.19 (1.04, 1.36)	1.2 (1.07, 1.36)	1.16 (0.99, 1.37)	1.21 (1.08, 1.34)	1.11 (0.94, 1.3)	1.24 (1.12, 1.39)	1.24 (1.1, 1.4)	1.15 (1.01, 1.32)
Lag 0	1.27 (1.12, 1.44)	1.03 (0.91, 1.18)	1.13 (0.99, 1.30)	1.14 (1.01, 1.29)	1.02 (0.85, 1.21)	1.19 (1.07, 1.33)	0.97 (0.82, 1.16)	1.22 (1.10, 1.36)	1.15 (1.01, 1.30)	1.13 (0.99, 1.30)
Lag 1	1.5 (1.33, 1.69)	1.4 (1.25, 1.57)	1.31 (1.15, 1.49)	1.55 (1.39, 1.73)	1.02 (0.86, 1.22)	1.62 (1.48, 1.78)	1.08 (0.92, 1.27)	1.63 (1.48, 1.79)	1.36 (1.21, 1.52)	1.55 (1.38, 1.74)
Lag 2	1.49 (1.32, 1.68)	1.44 (1.29, 1.62)	1.42 (1.26, 1.61)	1.5 (1.34, 1.68)	1.3 (1.11, 1.52)	1.53 (1.39, 1.68)	1.32 (1.14, 1.53)	1.53 (1.39, 1.69)	1.5 (1.34, 1.67)	1.44 (1.27, 1.62)
Lag 3	1.16 (1.02, 1.32)	1.07 (0.94, 1.21)	1.06 (0.93, 1.21)	1.14 (1.02, 1.29)	1.05 (0.89, 1.23)	1.13 (1.01, 1.25)	1.05 (0.9, 1.23)	1.13 (1.02, 1.26)	1.1 (0.97, 1.24)	1.13 (0.99, 1.28)
Lag 4	1.04 (0.9, 1.19)	1.15 (1.02, 1.3)	1.06 (0.93, 1.22)	1.13 (1, 1.27)	1.01 (0.86, 1.2)	1.12 (1.01, 1.25)	0.98 (0.83, 1.15)	1.15 (1.03, 1.29)	1.07 (0.94, 1.21)	1.13 (1, 1.29)
Lag 5	1.22 (1.07, 1.38)	1.05 (0.93, 1.2)	1.05 (0.92, 1.2)	1.19 (1.06, 1.34)	1.08 (0.92, 1.27)	1.14 (1.03, 1.27)	1.1 (0.95, 1.29)	1.13 (1.01, 1.27)	1.07 (0.94, 1.21)	1.2 (1.06, 1.36)
Lag 6	1.01 (0.88, 1.16)	1.06 (0.93, 1.2)	1.02 (0.89, 1.18)	1.05 (0.92, 1.18)	0.94 (0.78, 1.12)	1.07 (0.96, 1.2)	0.95 (0.8, 1.12)	1.08 (0.96, 1.2)	0.98 (0.86, 1.12)	1.1 (0.96, 1.25)
Lag 7	1.1 (0.97, 1.26)	0.87 (0.76, 1)	0.97 (0.85, 1.12)	0.98 (0.86, 1.11)	0.92 (0.77, 1.09)	1 (0.89, 1.11)	0.88 (0.74, 1.04)	1.02 (0.91, 1.14)	0.94 (0.83, 1.07)	1.02 (0.89, 1.17)

Note: 95% confidence intervals shown in parentheses.

Figure S1. Correlation matrix between neighborhood-level characteristics.

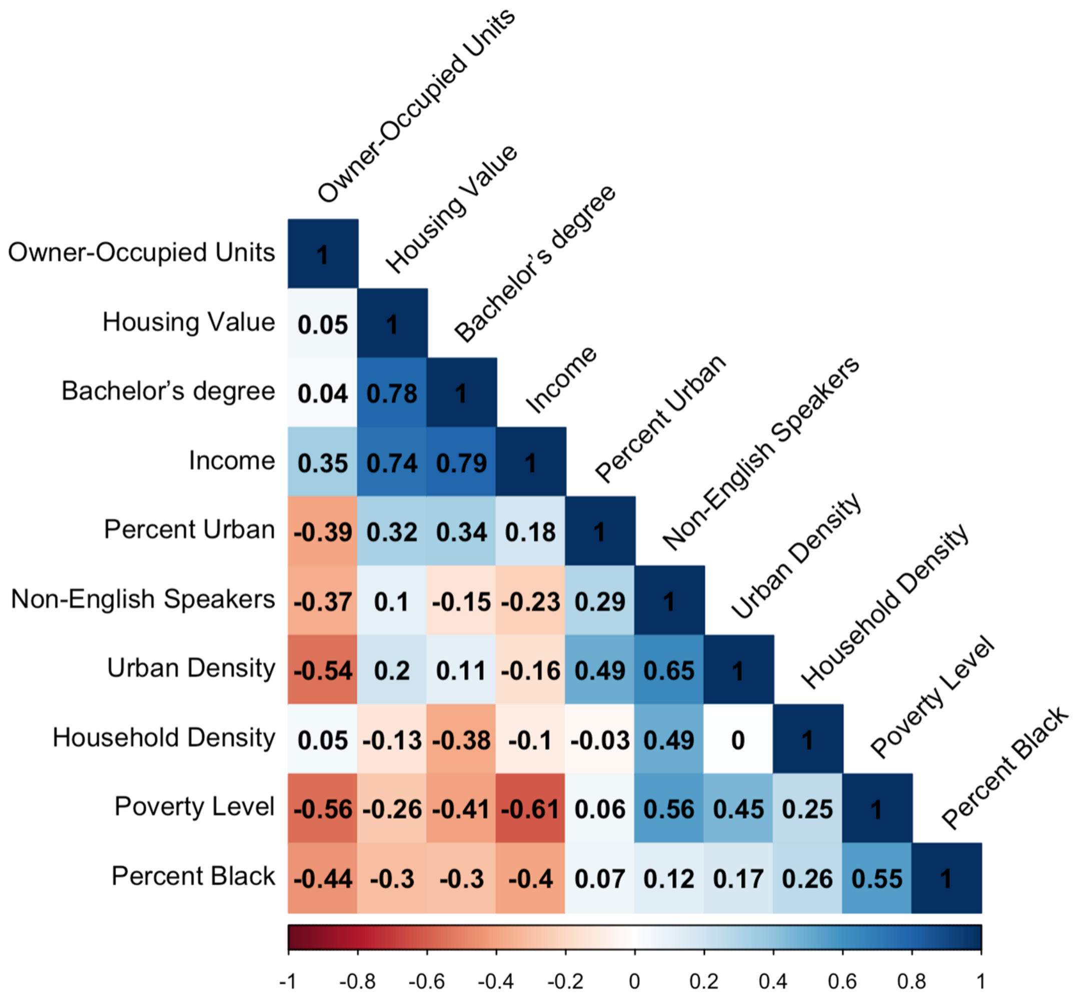

Figure S2. Visualization of matching procedure.

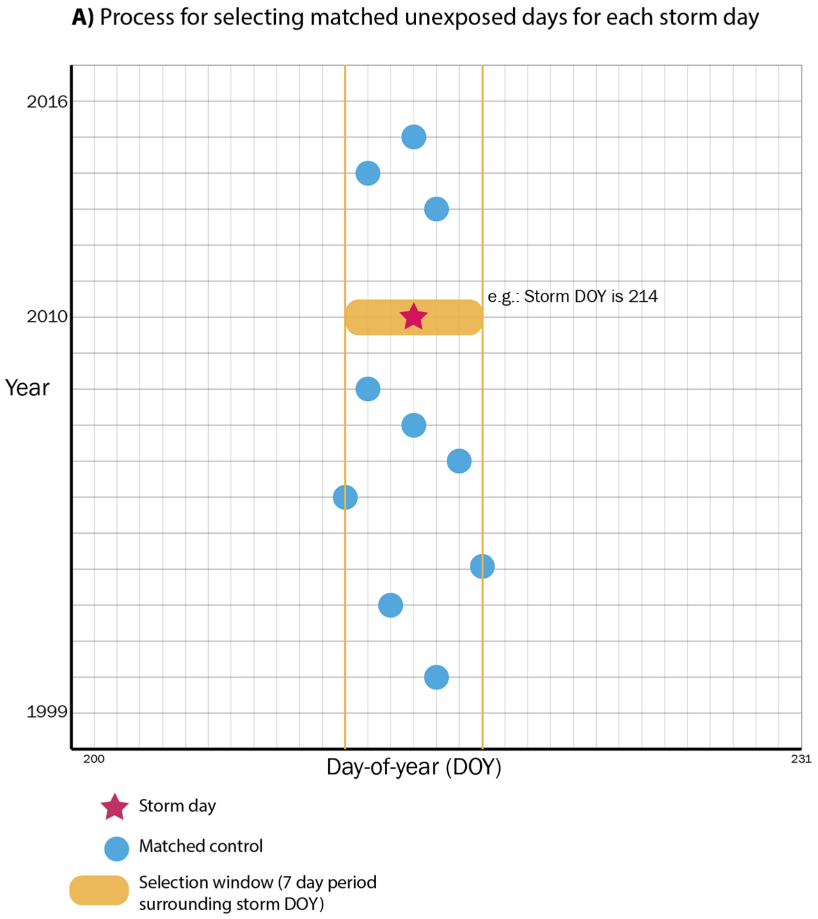

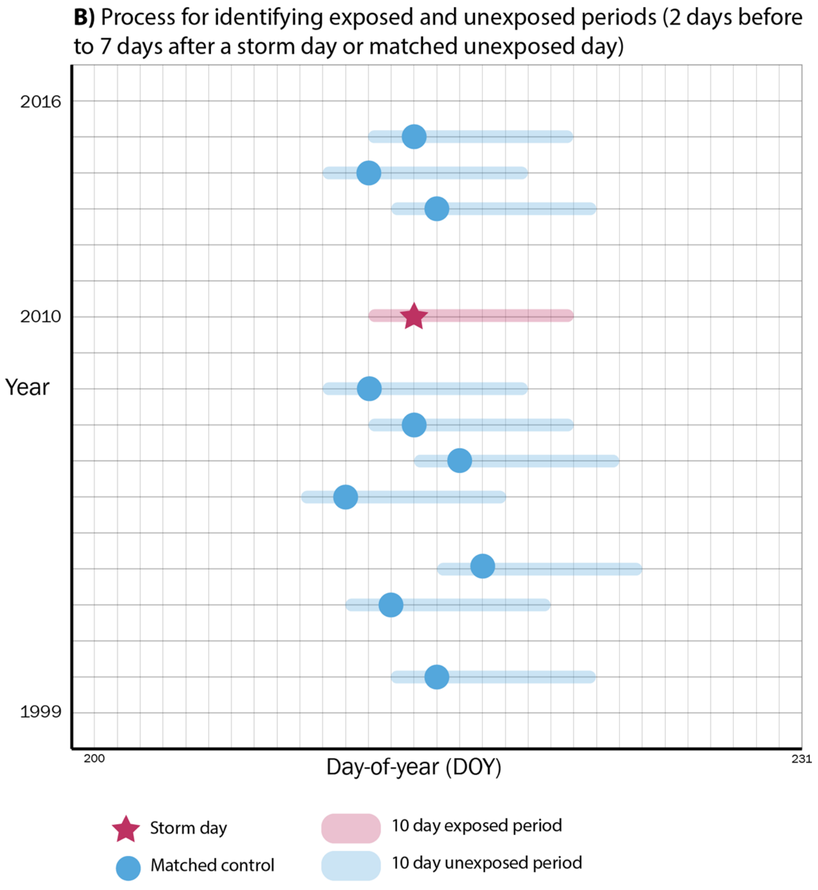

Supplement: Supplementary file 1 — Supplementary information [file 41467_2023_37675_MOESM1_ESM.pdf]
