# Supplementary material for: Health disparities among older adults following tropical cyclone exposure in Florida
Source: Nat Commun. 2023 Apr 19;14:2221. doi: 10.1038/s41467-023-37675-7 (PMC10115860; doi:10.1038/s41467-023-37675-7)
Supplement: Supplementary file 2 — Reporting Summary [file 41467_2023_37675_MOESM2_ESM.pdf]

nature portfolio

Corresponding author(s): Kate Burrows
Last updated by author(s): Jan 12, 2023

Reporting Summary

Nature Portfolio wishes to improve the reproducibility of the work that we publish. This form provides structure for consistency and transparency in reporting. For further information on Nature Portfolio policies, see our Editorial Policies and the Editorial Policy Checklist.

Statistics

For all statistical analyses, confirm that the following items are present in the figure legend, table legend, main text, or Methods section.

n/a	Confirmed	
☐	☒	The exact sample size (n) for each experimental group/condition, given as a discrete number and unit of measurement
☐	☒	A statement on whether measurements were taken from distinct samples or whether the same sample was measured repeatedly
☐	☒	The statistical test(s) used AND whether they are one- or two-sided<br>*Only common tests should be described solely by name; describe more complex techniques in the Methods section.*
☐	☒	A description of all covariates tested
☐	☒	A description of any assumptions or corrections, such as tests of normality and adjustment for multiple comparisons
☐	☒	A full description of the statistical parameters including central tendency (e.g. means) or other basic estimates (e.g. regression coefficient) AND variation (e.g. standard deviation) or associated estimates of uncertainty (e.g. confidence intervals)
☐	☒	For null hypothesis testing, the test statistic (e.g. F, t, r) with confidence intervals, effect sizes, degrees of freedom and P value noted<br>*Give P values as exact values whenever suitable.*
☒	☐	For Bayesian analysis, information on the choice of priors and Markov chain Monte Carlo settings
☒	☐	For hierarchical and complex designs, identification of the appropriate level for tests and full reporting of outcomes
☒	☐	Estimates of effect sizes (e.g. Cohen's d, Pearson's r), indicating how they were calculated

Our web collection on statistics for biologists contains articles on many of the points above.

Software and code

Policy information about availability of computer code

Data collection	Data was collected from Medicare pay-per-services beneficiaries from 1999-2016.<br>Analyses were conducted using the R Statistical Software, version 3.6.3. Distributed lag nonlinear models were specified through the “dlnm” package (2.4.7).
Data analysis	We provide a sample of the code used in this project online at https://github.com/kateburrows/tropical_cyclone_NatureComm

For manuscripts utilizing custom algorithms or software that are central to the research but not yet described in published literature, software must be made available to editors and reviewers. We strongly encourage code deposition in a community repository (e.g. GitHub). See the Nature Portfolio guidelines for submitting code & software for further information.

Data

Policy information about availability of data

All manuscripts must include a data availability statement. This statement should provide the following information, where applicable:

- Accession codes, unique identifiers, or web links for publicly available datasets
- A description of any restrictions on data availability
- For clinical datasets or third party data, please ensure that the statement adheres to our policy

Exposure data are available through the “hurricaneexposure” package in R (https://cran.r-project.org/web/packages /hurricaneexposure/vignettes/hurricaneexposure.html). These data are based on tropical cyclones recorded in the HURDAT2 dataset (https://www.aoml.noaa.gov/

March 2021

hrd/hurdat/Data_Storm.html).
Health data (Medicare enrollees dynamic cohort) is available upon purchase and after an application process, from the Centers for Medicare & Medicaid Services (https://www.cms.gov/research-statistics-data-and-systems/cms-information-technology/accesstodataapplication).

Human research participants

Policy information about studies involving human research participants and Sex and Gender in Research.

Reporting on sex and gender	In addition to our primary analysis, we conducted a stratified analysis based on sex. We used sex rather than gender because sex is the available variable as collected in the Medicare dataset. We provide full results for these stratified models in Table S2.
Population characteristics	We obtained individual level data on sex, race, Medicaid dual-eligibility, and age. Summary statistics based on demographic characteristics are provided in Table 1 (Summary statistics for Medicare hospitalizations for included ZIPS (n=796) in Florida, 1999-2016). The majority of those hospitalized were White (87.9% of CVD hospitalizations and 87.7% of respiratory hospitalizations). More CVD and respiratory hospitalizations were for individuals 75-84 years of age (42.0% and 41.1%, respectively) compared to individuals 65-74 years of age (35.6% and 33.9% of hospitalizations) and those over 85 years (22.4% and 25.0% of hospitalizations). More women were hospitalized than men for both CVD (50.4%) and RD (55.9%).
Recruitment	Secondary data was obtained from the Medicare enrollees dynamic cohort and includes all pay-per-service Medicare beneficiaries (≥65 years) in Florida (1999-2016) for cardiovascular and respiratory disease hospitalizations. The authors did not do any recruitment.
Ethics oversight	Institutional Review Board at Yale University

Note that full information on the approval of the study protocol must also be provided in the manuscript.

Field-specific reporting

Please select the one below that is the best fit for your research. If you are not sure, read the appropriate sections before making your selection.

☐ Life sciences ☒ Behavioural & social sciences ☐ Ecological, evolutionary & environmental sciences

For a reference copy of the document with all sections, see nature.com/documents/nr-reporting-summary-flat.pdf

Behavioural & social sciences study design

All studies must disclose on these points even when the disclosure is negative.

Study description	In this study, we used a mixed effect Poisson regression model to estimate the relative risk (RR) of hospitalizations for tropical cyclone periods compared to unexposed periods, incorporating a distributed lag for storm exposures
Research sample	Data was obtained from the Medicare enrollees dynamic cohort and includes all pay-per-service Medicare beneficiaries (≥65 years) in Florida (1999-2016) for cardiovascular and respiratory disease hospitalizations. The majority of those hospitalized were White (87.9% of CVD hospitalizations and 87.7% of respiratory hospitalizations). More CVD and respiratory hospitalizations were for individuals 75-84 years of age (42.0% and 41.1%, respectively) compared to individuals 65-74 years of age (35.6% and 33.9% of hospitalizations) and those over 85 years (22.4% and 25.0% of hospitalizations). More women were hospitalized than men for both CVD (50.4%) and RD (55.9%). Medicare beneficiaries were selected as the sample because they are largely representative of the population of older adults in America.
Sampling strategy	We did not perform any recruitment or sampling for this study. We relied solely on the Medicare enrollees dynamic cohort (secondary administrative data). In this study, we used data from 1999-2016 in the state of Florida, and obtained cardiovascular and respiratory disease hospitalizations for all pay-per-services Medicare beneficiaries (aged 65 or older).
Data collection	No data was collected in this study. We relied solely on the Medicare enrollees dynamic cohort (secondary administrative data of hospitalization records).
Timing	We obtained all hospitalization records from 1999-2016.
Data exclusions	We conducted a complete case analysis on participants living in ZCTAs that experienced at least one tropical cyclone during the study period.
Non-participation	We did not collect any primary data in this study, and relied only on secondary administrative data from the Medicare enrollees dynamic cohort. Overall, Medicare is highly representative of the older adults in American, as 98% of Americans aged 65 years or older are insured by Medicare (Mues et al., 2017). Our data includes all pay-for-service hospitalizations (Part A) and does not include beneficiaries who are enrolled in Medicare Advantage (Part C) (for whom claims are processed separately).

Mues, K. E., Liede, A., Liu, J., Wetmore, J. B., Zaha, R., Bradbury, B. D., Collins, A. J., & Gilbertson, D. T. (2017). Use of the Medicare database in epidemiologic and health services research: a valuable source of real-world evidence on the older and disabled populations in the US. Clinical epidemiology, 9, 267-277.

Randomization

No randomization occurred in this study. In our Poisson regression we controlled for day of week, year, and the number of Medicare beneficiaries. We also account for clustering at the zip code level by including a random intercept.
We also examined stratified models by individual (age, sex, and Medicaid dual eligibility) and community characteristics (urbanicity, level of education, level of income and poverty, racial composition, and English speaking).

Reporting for specific materials, systems and methods

We require information from authors about some types of materials, experimental systems and methods used in many studies. Here, indicate whether each material, system or method listed is relevant to your study. If you are not sure if a list item applies to your research, read the appropriate section before selecting a response.

Materials & experimental systems

n/a	Involved in the study	
☒	☐	Antibodies
☒	☐	Eukaryotic cell lines
☒	☐	Palaeontology and archaeology
☒	☐	Animals and other organisms
☒	☐	Clinical data
☒	☐	Dual use research of concern

Methods

n/a	Involved in the study	
☒	☐	ChIP-seq
☒	☐	Flow cytometry
☒	☐	MRI-based neuroimaging
